# Experimental and numerical simulation studies on the mechanical properties and failure characteristics of rock masses with weak interlayers

**DOI:** 10.1371/journal.pone.0327047

**Published:** 2025-08-07

**Authors:** Feng Wang, Yong Yang, Yuchi Wang, Zhaoyu Li, Baoliang Han, Yang Bin, Jun Hu, Yujiang Yang, Zhiguo Xia

**Affiliations:** 1 Open-pit Mining Branch of Ansteel Mining Gongchangling Co., Ltd., Liaoyang, Liaoning, China; 2 School of Civil Engineering, University of Science and Technology Liaoning, Anshan, China; 3 School of Mining Engineering, University of Science and Technology Liaoning, Anshan, China; Manipal Academy of Higher Education, INDIA

## Abstract

To elucidate the mechanical properties and failure behaviors of rock-like materials with weak interlayers of varying inclinations and thicknesses, uniaxial compression tests were conducted on such rock-like materials. The effects of interlayer inclination and thickness on the acoustic emission ringing counts and macroscopic fracture of the rock-like materials were investigated. From a mesoscopic perspective, the crack initiation and propagation processes, stress field distribution characteristics, and energy evolution laws of the rock-like materials with weak interlayers were analyzed. Additionally, the failure modes obtained from the experiments were compared with those from numerical simulations. The results indicate that as the interlayer thickness or inclination increases, the peak strength and elastic modulus of the specimens gradually decrease. Specifically, under the influence of interlayer thickness, the peak strength and elastic modulus decrease by 38.27% and 68.69%, respectively, while under the influence of interlayer inclination, they decrease by 51.28% and 8.47%, respectively. The energy dissipation of the specimens is mainly concentrated in the post-peak stage and is closely related to the propagation and coalescence of microcracks within the rock mass. The initial failure typically occurs at the weak interlayer or at the interface between layers. The weak interlayer serves as the primary zone for microcrack initiation, and the stress concentration zones are mainly distributed on the upper and lower sides of the interlayer. The failure mode transitions gradually from tensile failure to shear failure, ultimately dominated by a combined tensile-shear failure. Moreover, the failure primarily manifests as the overall failure of the specimens with weak interlayers.

## 1 Introduction

**Fig 1:**
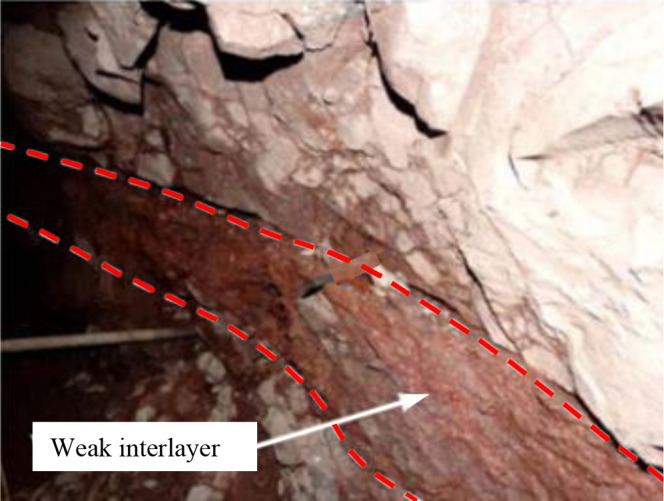
Composite rock mass containing weak interlayers [11].

Natural rock mass is a geological body with complex mechanical properties, often containing numerous discontinuities such as fissures, joints, and weak interlayers [1–3]. Among these, weak interlayers pose a significant threat to the stability of deep underground engineering and slopes due to their unique mechanical characteristics, such as low strength, low elastic modulus, and high Poisson’s ratio. When weak interlayers form a composite entity with hard and brittle rocks (as shown in Fig 1), the significant differences in mechanical properties between the two result in highly uneven stress distribution within the composite rock mass, which can lead to stability issues such as collapses, rockbursts, and cracking [4–6]. Therefore, conducting in-depth research on the strength, deformation characteristics, and mechanical properties of composite rock masses containing weak interlayers is of great theoretical significance and practical value for ensuring the safety and stability of underground engineering and slopes [7–10].

Despite extensive and in-depth research conducted by numerous scholars on the mechanical properties and failure characteristics of composite rock masses containing weak interlayers, there are still some deficiencies and limitations in existing studies [12–14]. Xu et al. [15] classified the main failure modes of rock masses containing weak interlayers into tensile cracking failure, spalling, and contact shear-slip failure based on the rock mass structure control theory. Liang et al. [16] studied composite rock masses composed of anhydrite and rock salt and found that the overall strength of the specimens depends on the weaker component in the composite rock mass. Tien et al. [17] explored the relationship between the inclination angle of weak interlayers and the strength and elastic modulus of composite rocks, and established a failure criterion that reflects the effect of the inclination angle of weak interlayers. Although these studies have revealed the influence of weak interlayers on the mechanical behavior of composite rock masses, research on the specific effects of the two key parameters, namely the thickness and inclination angle of weak interlayers, on composite rock masses is still insufficient, especially regarding the mechanical response and failure behavior under the coupled effects of these two parameters.

In the process of exploring the failure mechanisms of composite rock masses, the energy evolution mechanism is an important factor that cannot be ignored [18,19]. However, existing studies often overlook the analysis of the energy evolution mechanism when discussing the failure process of composite rock masses containing weak interlayers, or they are limited to the study of a single variable. Jia et al. [20] used the PFC particle flow code to investigate the combined effects of strength ratio and soft layer thickness on the microcrack evolution mechanism in layered rocks, finding that cracks in the hard layer are mainly vertical and near-vertical tensile failures, while cracks in the soft layer are mainly shear failures. Han et al. [21] took the interlayer shear weak zones of the Baihetan Hydropower Station as the research object and established a two-dimensional geological numerical model using PFC2D to investigate the effects of filling rate, joint roughness coefficient, and normal stress on the shear characteristics of interlayer shear weak zones. However, this study also failed to fully reflect the synergistic effects of the thickness and inclination angle of weak interlayers on the failure mechanisms of composite rock masses. When a material is subjected to external loads, the elastic strain energy stored within it is partially released with changes in the microstructure, forming acoustic emission signals [22–24]. Xie et al. [25] studied the characteristics of the acoustic emission RA-AF values during the failure process of rock-like materials with alternating hard and soft layers, demonstrating that both shear and tensile failures occur during the failure process of layered rock masses. However, these studies did not delve into the relationship between the energy release process and the failure mode, neglecting the analysis of the energy evolution mechanism.

In view of the above deficiencies and limitations, this study uses similar simulation materials to prepare composite rock masses containing weak interlayers. Through a combination of experiments and numerical simulations, the effects of the synergistic action of the thickness and inclination angle of weak interlayers on the stress-strain behavior, deformation characteristics, and failure modes of rock masses are systematically analyzed. The synergistic effects of the two key parameters, namely the thickness and inclination angle of weak interlayers, are comprehensively considered, and the energy evolution mechanism of composite rock masses during compression is explored. It is hoped that this study can provide a more comprehensive understanding of the mechanical properties and failure mechanisms of composite rock masses containing weak interlayers and offer theoretical references for the safety and stability of underground engineering.

## 2 Sample Materials and methods

### 2.1 Sample preparation

**Fig 2:**
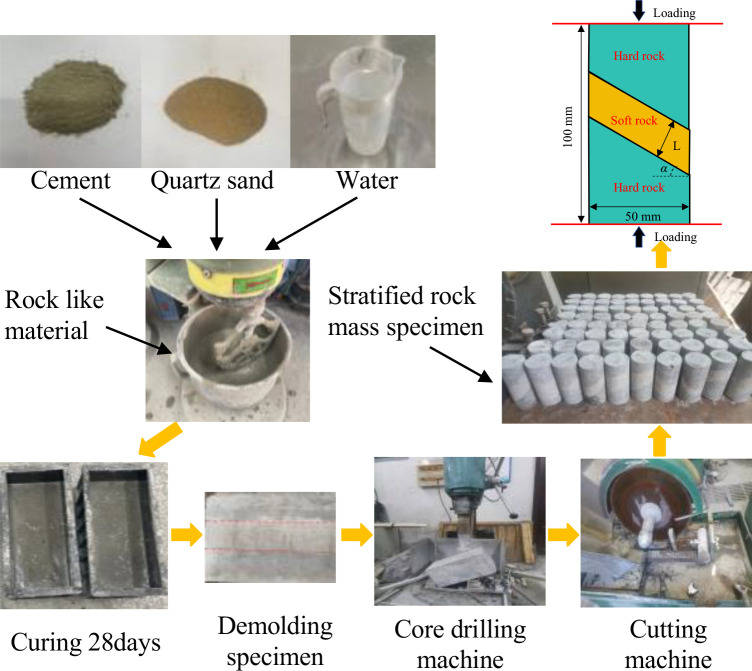
Sample preparation process.

Based on the similarity theory, composite rock mass samples containing weak interlayers were prepared using sand, cement, and water. The mixture ratio for the soft rock was cement:sand:water = 1:4:0.75, while for the hard rock, it was 3:1:1. The specific preparation process is illustrated in Fig 2: First, various materials were weighed according to the designed ratios and mixed uniformly. Subsequently, the uniformly mixed solid materials were placed into a mixer, and an appropriate amount of water was added to stir the mixture until it reached a specific fluidity. The prepared cement mortar was then poured into a cubic mold with dimensions of 150 mm × 150 mm × 150 mm in multiple batches. After the mortar had solidified to a certain strength but had not yet fully hardened, the next rock layer was poured, and vibrations were applied to eliminate air bubbles in the sample. Afterward, the prepared samples were immersed in water for curing for 28 days. A vertical rock drilling machine with a drill bit inner diameter of 50 mm was used for coring the samples, and the inclination angle of the interlayer was controlled using a tripod stand. Finally, tools such as a grinding machine and sandpaper were used to flatten the ends of the cylindrical samples.

**Fig 3:**
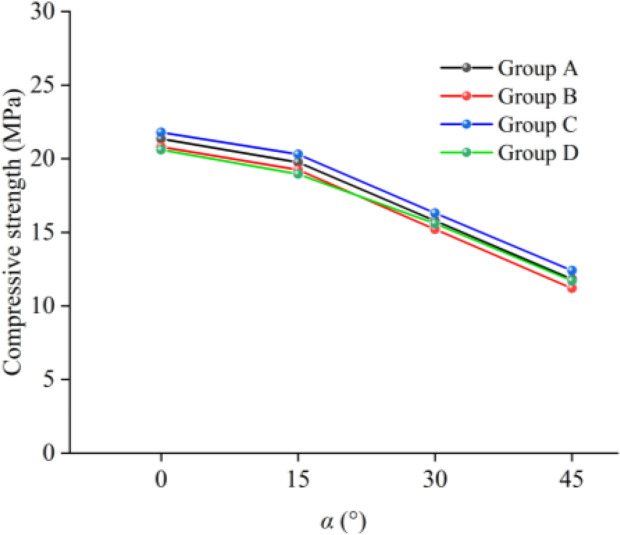
Compressive strength of samples with different inclination angles and the same thickness of weak interlayers (taking L = 20 mm as an example).

**Fig 4:**
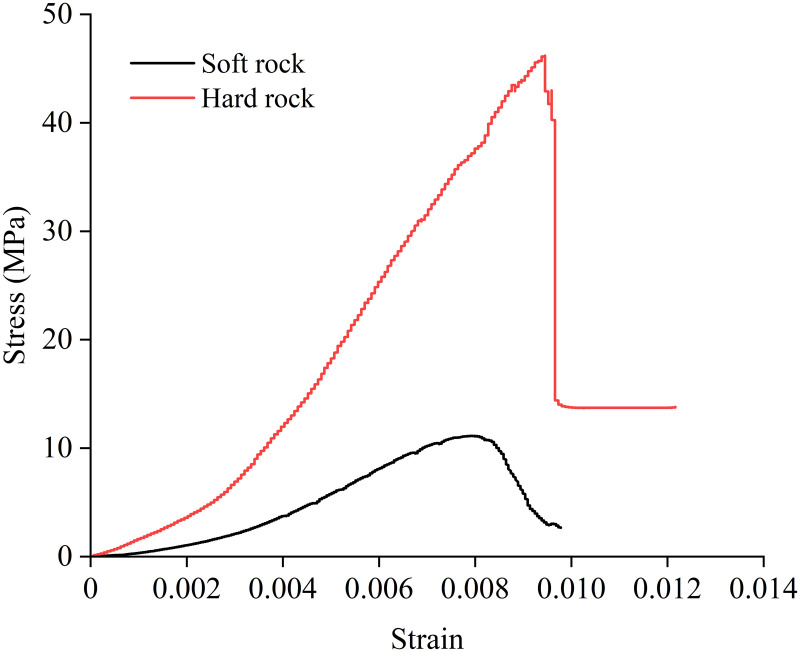
Stress-strain curve of soft and hard rock under uniaxial compression.

The laboratory tests were divided into two parts. A total of 4 different interlayer inclination angles (α) were set, namely 0°, 15°, 30°, and 45°. Four different interlayer thicknesses (L) were also set, specifically 10 mm, 20 mm, 30 mm, and 40 mm. Four samples were prepared for each group, considering the difficulty in achieving complete consistency in thickness and diameter during sample processing. By examining the compressive strengths of parallel samples at different inclination angles (taking L = 20 mm as an example, as shown in Fig 3), the samples with the best test results were selected as the analysis objects to prevent the occurrence of experimental accidents. According to the “Standard for Engineering Rock Mass Classification” (GB50218−94), rocks with a uniaxial compressive strength ≤ 30 MPa are defined as soft rocks. Based on this standard, the stress-strain curves for the soft and hard rock samples obtained through the mixture ratio tests are shown in Fig 4. The strength of the soft rock is approximately 11.11 MPa, while the strength of the hard rock is approximately 46.1 MPa. The physical and mechanical parameters of the soft and hard rock samples are listed in Table 1. As can be seen from Table 1, the density, peak strength, elastic modulus, and peak strain of the hard rock are all greater than those of the soft rock. The strength ratio of the hard rock to the soft rock samples is 4.15.

**Table 1:** Basic physical and mechanical parameters of rocklike material.

Rock type	Density (g/cm³)	Peak strength (MPa)	Elastic modulus (GPa)	Peak strain (10^ − 3^)
Soft rock	2.03	11. 11	2.25	7.89
Hard rock	2.10	45.91	6.47	9.40

### 2.2 Specimen preparation

**Fig 5:**
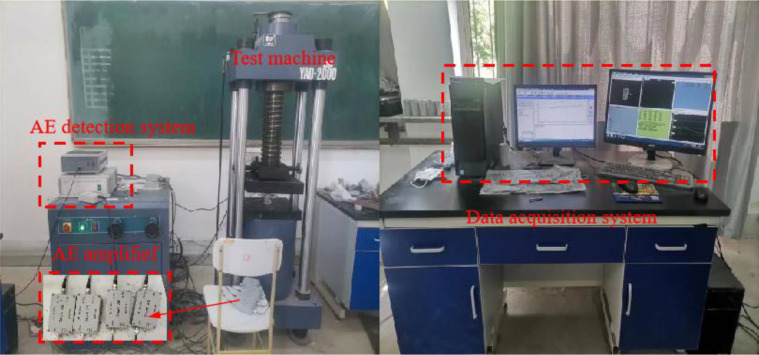
Uniaxial testing system.

The equipment used in the experiment includes uniaxial loading equipment and acoustic emission (AE) detection equipment, as shown in Fig 5. A YAD-2000 electro-hydraulic servo pressure testing machine produced by Changchun Kexin Test Instrument Co., Ltd. was used for the uniaxial compression tests. The testing equipment can provide a load of 2000 kN, with a displacement loading method at a rate of 0.1 mm/min. A DS2 series full-information AE signal analyzer produced by Beijing Soft Island Technology Co., Ltd. was used to monitor the failure process of the samples in real-time. The gain of the preamplifier for the AE signals was set to 40 dB, the monitoring threshold was set to 40 dB, the peak definition time (PDT) was set to 100 μs, the hit definition time (HDT) was set to 200 μs, and the hit lock time (HLT) was set to 300 μs. The sampling frequency was set to 3 MHz. Two sets of Smart AE preamplifiers and RS-2A sensors were orthogonally arranged 20 mm above and below the upper and lower end faces of the samples, with a frequency range set to 50 ~ 400 kHz and a resonant frequency of 150 kHz. The two units in each set of sensors were symmetrically distributed on the same plane, and silicone grease was used as a coupling agent to reduce the acoustic impedance mismatch between the sensors and the sample contact interface, thereby minimizing energy reflection losses at the interface and improving the transmission efficiency and monitoring accuracy of the AE signals. The sensors were gently fixed with rubber bands to increase the coverage area on the samples [24].

### 3.1 Mechanical properties

**Fig 6:**
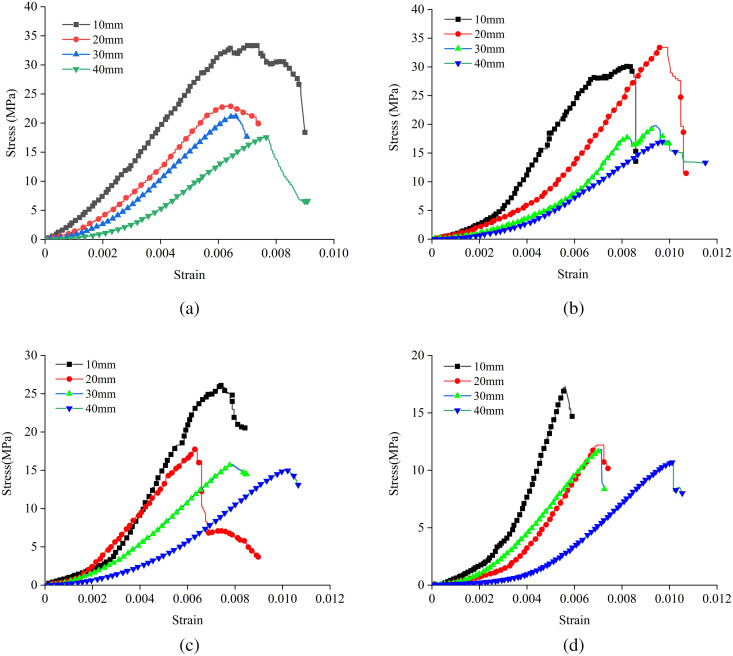
Stress-strain curves at different interlayer thicknesses (L) and inclination angles (α): (a) Interlayer inclination angle α = 0°, (b) Interlayer inclination angle α = 15°, (c) Interlayer inclination angle α = 30°, (d) Interlayer inclination angle α = 45°.

**Fig 7:**
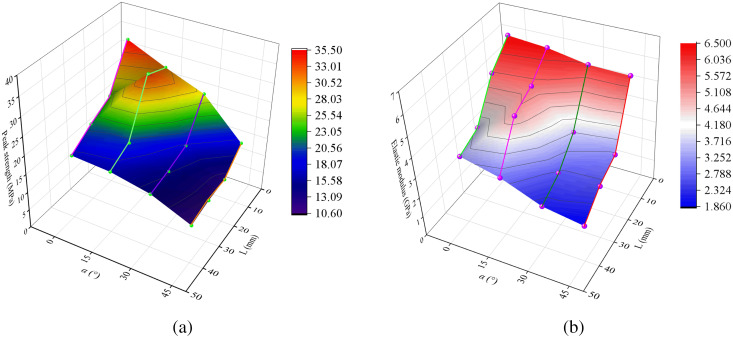
(a) Peak stress and (b) elastic modulus at different interlayer thicknesses (L) and inclination angles (α).

Figs 6 and 7 show the stress-strain curves of samples with different interlayer thicknesses at various interlayer inclination angles, as well as the peak strength and elastic modulus values of samples with different L and α, respectively. It can be observed from the figures that the stress-strain curves of all samples are generally similar, all going through the stages of pore compaction, elastic deformation, plastic deformation, and failure.

When the interlayer inclination angle (α) is constant, the peak strength and elastic modulus of the samples decrease with an increase in the interlayer thickness (L). This indicates that samples with thicker interlayers have lower bearing capacities and are more prone to failure. Additionally, as the interlayer thickness increases, the range of stress concentration areas within the rock mass gradually expands, leading to an increase in the damage range of the fracture surface and an increase in strain values with increasing L. It is worth noting that for the sample with α = 15° and L = 20 mm, the interlayer and the hard rock together form a strong shear plane, enhancing the overall shear strength of the rock mass and resulting in relatively high strength. Taking the samples with an interlayer inclination angle of 45° as an example, as shown in Fig 7, when L ranges from 10 mm to 40 mm, the peak strength decreases from 17.30 MPa to 10.68 MPa, a reduction of 38.27%, and the elastic modulus decreases from 5.94 GPa to 1.86 GPa, a reduction of 68.69%. This indicates that the interlayer thickness has a significant impact on both the uniaxial compressive strength and elastic modulus of the samples, with a greater effect on the elastic modulus.

When L is constant, the peak strength and elastic modulus of the samples generally show a decreasing trend with an increase in the inclination angle of the weak interlayer, and the interlayer slip effect becomes more pronounced, gradually weakening the deformation capacity of the samples as the interlayer inclination angle increases. When the interlayer inclination angle (α) is 0°, the shear stress has a relatively small effect on the interlayer interface, suppressing shear slip within the rock mass. Under the conditions of α = 15° and L = 30 mm, the elastic modulus of the sample exhibits certain abnormal fluctuations due to the synergistic effect between the interlayer and the surrounding rock. Additionally, taking the samples with an interlayer thickness of 10 mm as an example, when α ranges from 0° to 45°, the peak strength decreases from 35.5 MPa to 17.3 MPa, a reduction of 51.28%, and the elastic modulus decreases from 6.49 GPa to 5.94 GPa, a reduction of 8.47%. This indicates that the interlayer inclination angle has a relatively significant effect on the peak strength.

### 3.2 Acoustic emission activity

**Fig 8:**
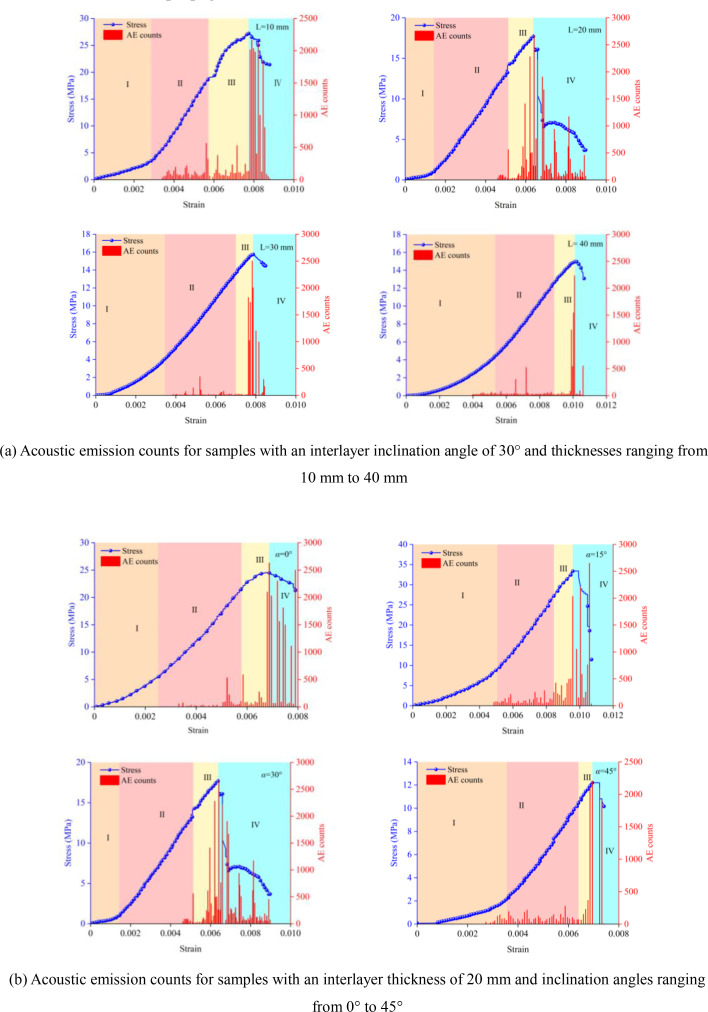
Stress-strain curves and acoustic emission counts for samples with different weak interlayer thicknesses and inclination angles. **(a)** Acoustic emission counts for samples with an interlayer inclination angle of 30° and thicknesses ranging from 10 mm to 40 mm. **(b)** Acoustic emission counts for samples with an interlayer thickness of 20 mm and inclination angles ranging from 0° to 45°.s.

Fig 8 shows the stress-strain curves and AE counts for samples with different interlayer thicknesses at an interlayer inclination angle (α) of 30° and for samples with different interlayer inclination angles at an interlayer thickness (L) of 20 mm. It can be observed from the figure that the acoustic emission signals exhibit distinct stage characteristics. In the initial compaction stage (Stage I), the micro-pores within the samples are gradually compacted, but there is basically no crack generation during this process, so the number of acoustic emission events is essentially zero. This indicates that the AE counts in this stage are not affected by the interlayer inclination angle and thickness. In the elastic deformation stage (Stage II), although there are some acoustic emission events, the number of events is relatively small because the micro-cracks in the samples are in the initial stages of development. In the elasto-plastic deformation stage (Stage III), as the stress increases, new micro-cracks are generated, and the existing micro-cracks continue to propagate, leading to an accelerated increase in the AE counts. When the samples reach the failure deformation stage (Stage IV), the stress-strain curve shows a significant drop, and large-scale fractures occur in the hard rock areas of the samples. Macroscopic cracks penetrate through the interlayer and interconnect, gradually leading to the failure of the samples, and the AE counts increase significantly. Under the continuous action of the load, the weak interlayer with a certain inclination angle undergoes progressive slippage, ultimately resulting in the overall failure of the specimens.

Under the action of external loads, cracks first appear and propagate in the weak interlayer with lower strength, causing the stress-strain curve to exhibit certain fluctuations before reaching the peak stress. As the loading continues, the fractured zone in the weak interlayer forms a linked fracture with the nearby hard rock. However, the presence of the weak interlayer offsets some of the compressive-shear effects and is accompanied by shear slippage, causing the stress to transition from a slow and steady decrease to a staged sharp decrease. Based on this, the stability of rocks containing weak interlayers can be assessed by combining the acoustic emission characteristics of samples with different interlayer inclination angles and analyzing the characteristics of crack propagation and coalescence.

## 4 Numerical simulation

### 4.1 Numerical model and parameter calibration

**Fig 9:**
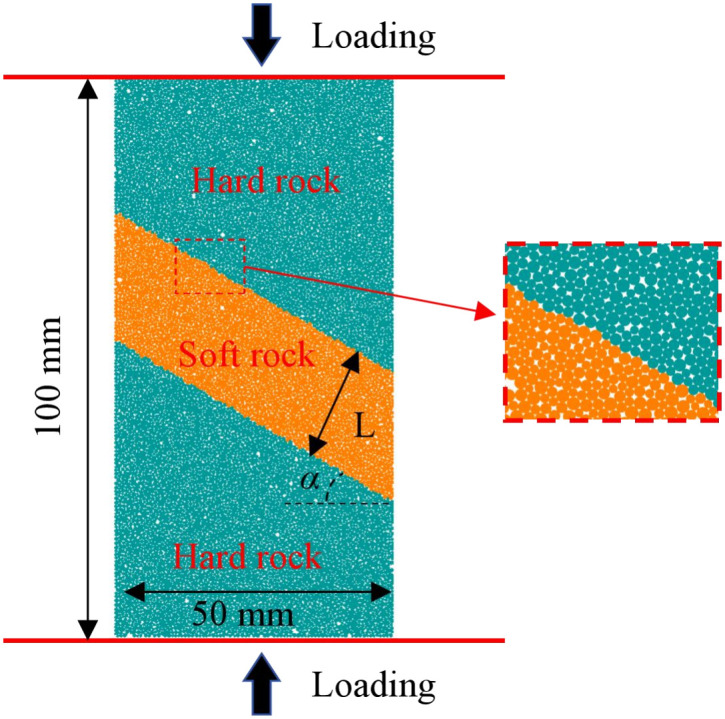
Model establishment in PFC2D.

**Fig 10:**
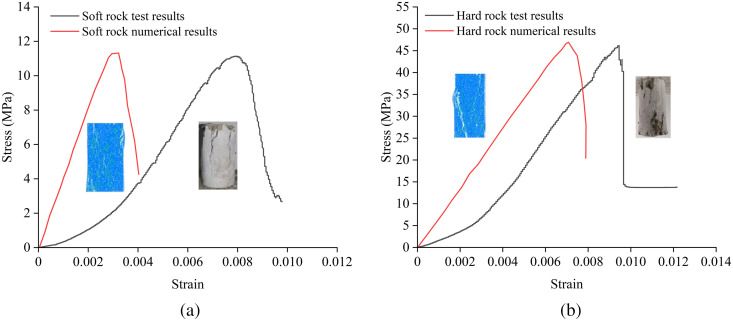
Comparison between the experimental results and the numerical simulation results. **(a)** Soft rock, **(b)** Hard rock.

**Fig 11:**
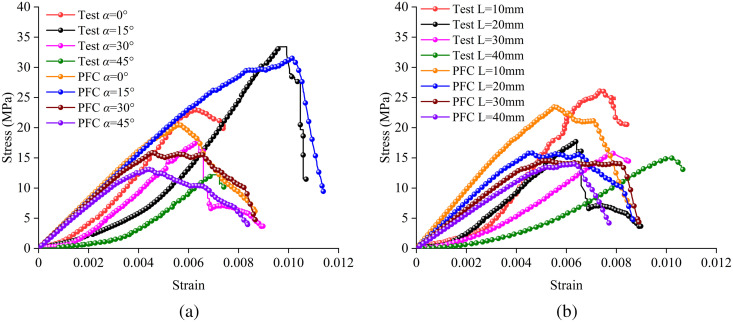
Comparison between experimental results and numerical simulations: (a) Comparison of different α values at L = 20 mm, (b) Comparison of different L values at α = 30°.

The discrete element method using the two-dimensional particle flow code (PFC2D) can effectively investigate the evolution process of micro-cracks within composite rock masses containing weak interlayers under compression. Among the available models, the linear parallel bond model can more accurately simulate the mechanical behavior and bonding effects of rock mass materials [26,27]. Therefore, in this study, a two-dimensional rectangular plane strain model with the same dimensions as the physical experiments (50 mm × 100 mm) was established, as shown in Fig 9. The particle radius distribution in the model ranges from 0.25 mm to 0.42 mm, and each model contains approximately 12,673 particles and 27,007 contact relationships. Based on laboratory tests, a large number of numerical simulation tests were conducted under conditions similar to those of the laboratory tests. Subsequently, the numerical simulation results were compared with the laboratory test results. The micro-mechanical parameters of the soft and hard rocks were repeatedly adjusted and compared using the “trial-and-error” method, and the results are presented in Table 2. Fig 10 shows the comparison results of the stress-strain curves and failure modes between the laboratory tests and numerical simulations for the soft and hard rocks. As can be seen from Fig 10, the failure modes of the samples obtained from the laboratory tests and numerical simulations show good consistency, and the peak strengths and elastic moduli are basically the same. Additionally, Fig 11 further compares the stress-strain curves of composite rock masses containing weak interlayers under different interlayer thicknesses (L) and inclination angles (α). Since the particles in the PFC simulation software are rigid bodies, the initial compaction stage is not reflected in the stress-strain curves, resulting in slightly smaller peak strains in the numerical simulations compared to the laboratory test results. However, the elastic moduli, uniaxial compressive strengths, and stress-strain evolution curves are basically consistent, indicating that the PFC2D numerical simulation results can well reveal the macro-mechanical properties of composite rock masses containing weak interlayers.

**Table 2:** Microscopic parameters for composite rock masses with weak interlayers in PFC2D.

Hard rock		Soft rock	
Parameter	value	Parameter	value
Minimum radius (mm)	0.25	Minimum radius (mm)	0.25
Ratio of radius	1.66	Ratio of radius	1.66
Density of the particle (kg/m3)	2480	Density of the particle (kg/m3)	2400
Contact modulus of particles (GPa)	3.6	Contact modulus of particles (GPa)	1.95
Contact bond spacing (mm)	0.05	Contact bond spacing (mm)	0.05
Porosity	0.1	Porosity	0.1
Friction angle of parallel key (°)	40	Friction angle of parallel key (°)	36
Parallel bond tensile strength (MPa)	3.10	Parallel bond tensile strength (MPa)	2.1
Local damping	0.5	Local damping	0.5
Cohesion of the parallel bond (MPa)	16.1	Cohesion of the parallel bond (MPa)	10.3

### 4.2 Micro-crack evolution process

**Fig 12:**
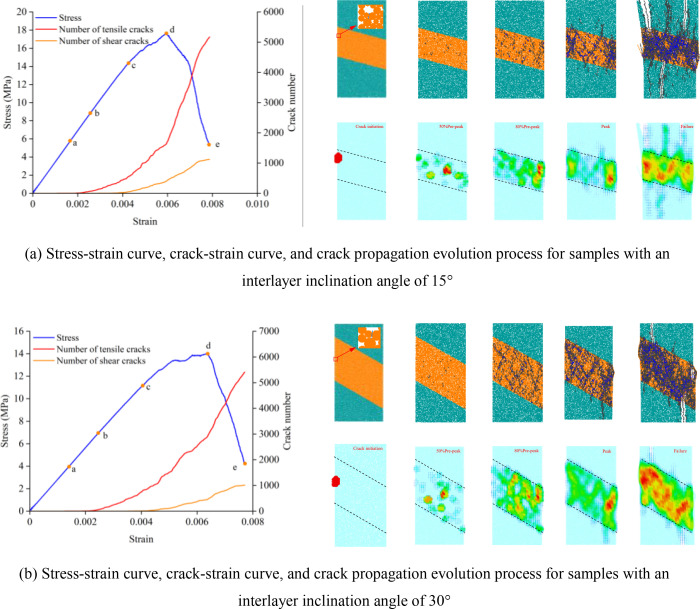
Stress-strain curves, crack-strain curves, and crack propagation evolution processes: (a) α = 15°, L = 30 mm, (b) α = 30°, L = 40 mm. **(a)** Stress-strain curve, crack-strain curve, and crack propagation evolution process for samples with an interlayer inclination angle of 15°. **(b)** Stress-strain curve, crack-strain curve, and crack propagation evolution process for samples with an interlayer inclination angle of 30°.

Fig 12 shows the micro-crack propagation and crack heat map evolution processes in composite rock masses containing weak interlayers under different inclination angles (α) and thicknesses (L). In the figure, tensile cracks are displayed in black, shear cracks in blue, and the redder the color in the crack heat map, the higher the number of cracks per unit area. It can be observed from the figure that the crack propagation process is divided into four stages. (1) Undamaged stage (0a stage), where no cracks are present inside the sample. (2) Stable crack growth stage (ac stage), during which micro-cracks begin to appear in the sample, and the number of cracks increases approximately linearly. (3) Unstable crack propagation stage (cd stage), where cracks inside the sample rapidly propagate, accompanied by the generation of new cracks, and a small number of macroscopic cracks gradually form in local areas. (4) Overall failure stage (de stage), where the stress rapidly decreases, micro-cracks gradually propagate and coalesce, increasing significantly, ultimately leading to the failure of the sample.

In this paper, the initial crack initiation stress (σci) is defined as the critical stress. Since the strength of the weak interlayer is lower than that of the hard rock, when the stress reaches σci (point a in Fig 12), cracks first appear in the weak interlayer. At this point, σci is 5.76 MPa and 4.00 MPa, respectively. In the ac stage, a large number of micro-cracks develop inside the weak interlayer, mainly tensile cracks. The damage accumulation caused by tensile action during this stage is significantly higher than that caused by shear action, indicating that the weak interlayer is the main initiation zone for micro-cracks. When the sample enters the cd stage, the existing cracks in the sample continue to propagate, accompanied by the generation of new cracks. At this point, the crack distribution inside the weak interlayer is relatively dense, and tensile cracks gradually extend into the hard rock areas. In the de stage of overall sample failure, the bearing capacity continues to decrease, and internal micro-cracks rapidly develop and accumulate. The development rate and number of tensile micro-cracks are much greater than those of shear micro-cracks. Due to the significantly reduced bearing capacity of the shear failure plane, stress concentration occurs in the boundary area between shear slip failure and non-shear slip failure, causing the hard rock portion to form a penetrating macroscopic tensile main crack, while shear slip failure gradually occurs inside the interlayer. The sample as a whole exhibits tensile-shear failure characteristics.

**Fig 13:**
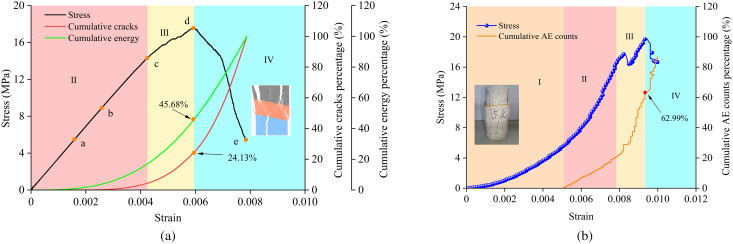
Comparison of failure processes between experiments and numerical simulations: (a) Numerical simulation of crack and energy evolution, (b) Evolution of acoustic emission counts in experiments.

Fig 13 presents a comparison of the evolution of numerically simulated cracks and acoustic emission (AE) counts from energy tests. As can be seen from the figure, before the stress-strain curve enters the elastoplastic stage, the number of micro-cracks is relatively small, and the AE event count remains at a low level. The elastic strain energy and input energy increase approximately linearly with the increase in axial strain, indicating that significant damage accumulation has not yet occurred inside the sample. As the stress approaches its peak and enters the failure stage, both the number of micro-cracks and the AE event count increase significantly, with most of the input energy being converted into dissipated energy. The cracks inside the sample accelerate their propagation, interconnect, and ultimately lead to failure. Additionally, the failure modes obtained from the experiments and numerical simulations are generally consistent, which further validates the effectiveness of numerical simulations in studying the fracture evolution process and failure mechanisms of composite rock masses.

**Fig 14:**
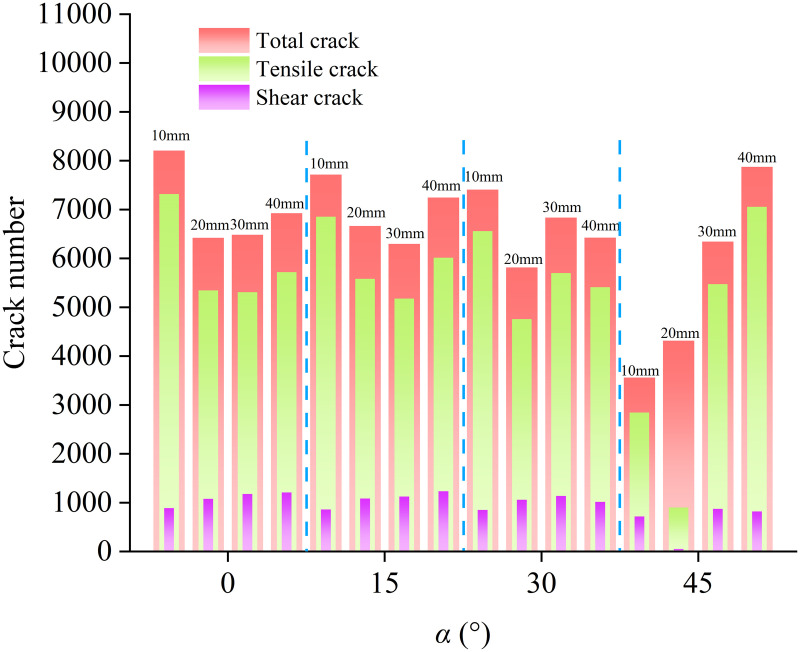
Variation in the total number of cracks in samples under different L and α.

Fig 14 shows the variation in the total number of cracks in samples under different inclination angles (α) and thicknesses (L) of interlayers. At the same interlayer inclination angle, as the interlayer thickness increases, the number of cracks in the samples exhibits different variation patterns. When the interlayer inclination angle is low (α = 0°, 15°), the number of cracks first decreases and then increases with increasing interlayer thickness, showing an overall decreasing trend. This is because the samples undergo tensile splitting failure when the interlayer thickness is small, and the failure mostly occurs in the hard rock, resulting in a larger number of cracks. When the interlayer inclination angle is 30°, the number of cracks fluctuates with increasing interlayer thickness but still shows an overall decreasing trend. When the interlayer inclination angle is 45°, the number of cracks gradually increases with increasing interlayer thickness. This is because when the interlayer thickness is small, the number of fractures inside the weak interlayer is relatively small during shear slip failure of the samples. As the interlayer thickness increases, more tensile cracks are also generated inside the weak interlayer during shear slip failure of the samples, leading to an increase in the number of cracks.

At the same interlayer thickness, when the interlayer thickness is 10 mm and the interlayer inclination angle is small, the cracks in the samples are widely distributed throughout the rock mass, mostly in the hard rock portion, resulting in a larger number of cracks. When the interlayer inclination angle increases to 45°, the number of cracks suddenly decreases, indicating that at this point, the samples are mainly subject to shear slip failure of the weak interlayer, resulting in a significant reduction in the number of fractures. When the interlayer thickness is 20 mm, the increased interlayer thickness causes the samples to still generate a relatively large number of tensile cracks during slip failure as the interlayer inclination angle increases, and the overall number of cracks shows a decreasing trend with increasing interlayer inclination angle. When the interlayer thickness is 30 mm or 40 mm, due to the larger interlayer thickness, although slip failure occurs as the interlayer inclination angle increases, tensile failure and tensile cracks are still generated, so the number of cracks does not decrease.

### 4.3 Stress field evolution characteristics

**Fig 15:**
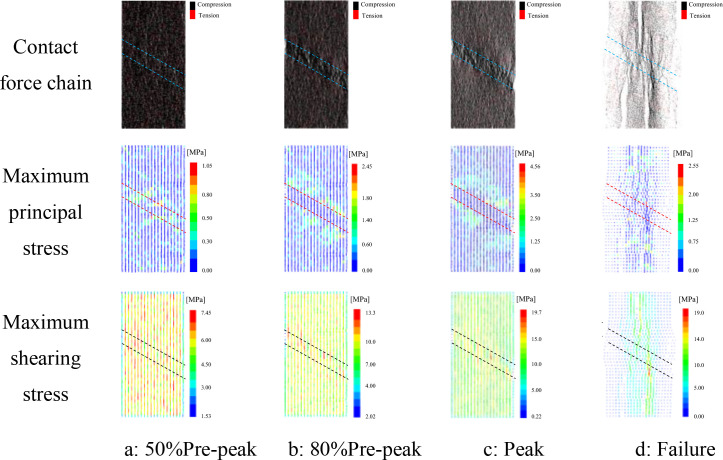
Stress field evolution (taking α = 30°, L = 10 mm as an example).

Fig 15 illustrates the stress field evolution process of a sample with α = 30° and L = 10 mm as an example. In the contact force chain diagram, black represents compressive chains, and red represents tensile chains. The thickness and density of the lines represent the magnitude of the contact forces. Thicker and denser lines indicate more intense compression between particles and larger contact forces. In the maximum principal stress diagram, tensile stress is positive, and compressive stress is negative. From the figure, it can be seen that the contact forces between particles first increase and then decrease after being compressed until failure occurs. This will cause the particles to move towards both sides of the failure plane and compress the particles near the failure plane.

Specifically, when the stress is in stage a, the contact force chains are mainly compressive, and tensile stress and maximum shear stress are uniformly distributed throughout the sample. The maximum principal stress concentration area mainly originates in the weak interlayer region, which is due to the lower strength of the weak rock compared to the hard rock. As the load continues to increase, the compression areas at both ends of the sample gradually extend towards the interior of the sample, eventually forming a compressive stress concentration band in the interlayer region inside the sample. The maximum principal stress expands from the interlayer interface towards both sides of the loading end, and the maximum shear stress moves towards the weak interlayer region. The maximum principal stress gradually increases to 4.56 MPa, and the maximum shear stress increases to 19.7 MPa. As the load increases again, the sample undergoes damage and failure, and the cracks propagate and coalesce. The compressive stress contact chains continuously break, forming tensile stress concentration areas. The maximum principal stress decreases to 2.55 MPa, and the maximum shear stress remains basically unchanged. The maximum stress in the sample runs through the sample, parallel to the loading direction. Ultimately, the sample exhibits a failure mode where vertical splitting and shear slip coexist.

### 4.4 Displacement field evolution characteristics

**Fig 16:**
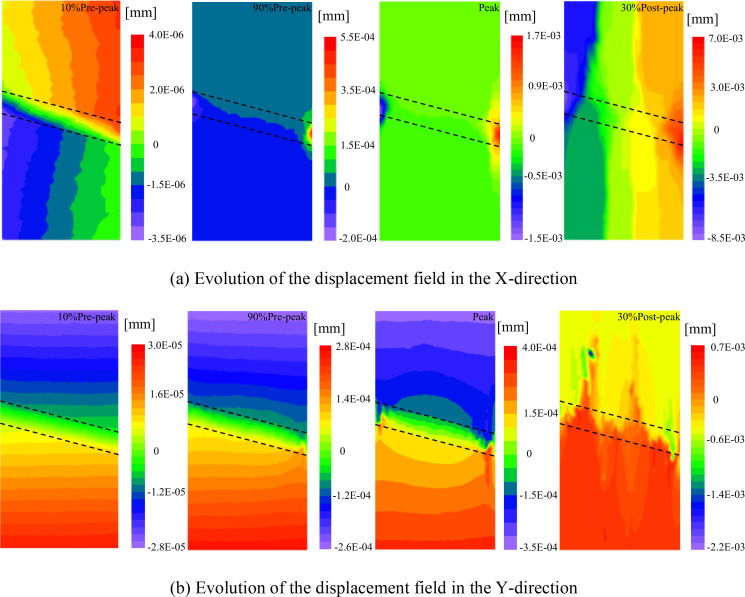
Displacement field evolution (taking α = 15°, L = 10 mm as an example). **(a)** Evolution of the displacement field in the X-direction. **(b)** Evolution of the displacement field in the Y-direction.

Fig 16 shows the displacement field evolution process for a sample with α = 30° and L = 10 mm. The displacement contour plot reflects the displacement of model particles, with the color intensity of the model representing the magnitude of particle displacement. The deformation direction along the X-axis is perpendicular to the loading direction, and the deformation direction along the Y-axis is parallel to the loading direction. Negative displacement indicates that the displacement direction is opposite to the positive direction of the X-axis and Y-axis.

During the initial loading, the maximum displacement values in the X-direction are distributed in the upper-right and lower-left corners of the sample, decreasing gradiently towards the other side. The displacement in the Y-direction in the interlayer region of the sample is close to zero, while the maximum Y-direction displacement values are at both ends of the sample, with opposite displacement directions. This is due to the loading stress applied at both ends of the sample, while the stress in the central region is weakened by the presence of the weak interlayer. The displacement increases with the increase in load. When the load reaches its peak, the X-direction displacement in the hard rock region and the middle of the interlayer is almost zero, and the ends of the interlayer become regions of concentrated displacement in opposite directions. This is because the hard rock provides symmetric restraint to the weak interlayer. As the load continues to increase, the X-direction displacements on both sides are almost symmetrically distributed in opposite directions, indicating that the sample undergoes tensile splitting failure. In summary, during the initial and later stages of loading, the Y-direction displacement field of the rock is relatively uniform, while the X-direction displacement field exhibits significant displacement gradient changes, indicating that the damage and failure of the rock are mainly controlled by tensile strain.

### 4.5 Energy variation characteristics

**Fig 17:**
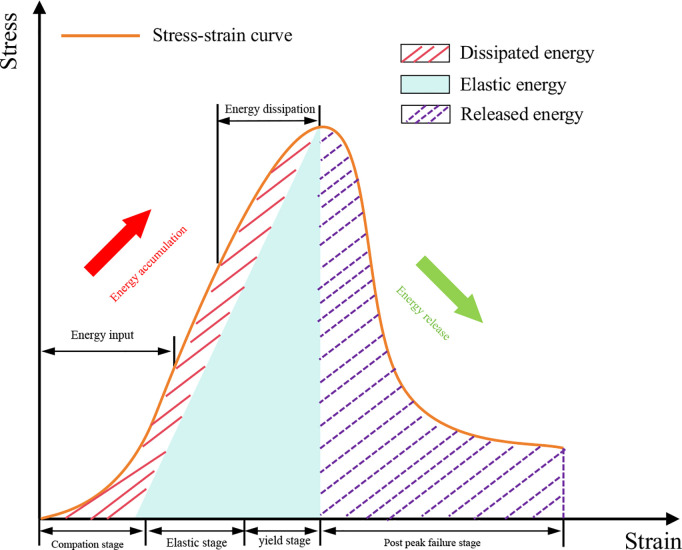
Energy evolution characteristics of rock under uniaxial compression.


The failure process of rock can be regarded as a process of energy accumulation and dissipation, where changes in internal energy lead to failure and instability. Analyzing the deformation behavior of rock using energy theory can effectively reveal the mechanism of rock deformation and failure [28,29], as shown in Fig 17. According to the first law of thermodynamics, the following relationship is obtained [30]:

$$U=U_{e}+U_{d}$$ (1)

In the formula, U represents the input strain energy, Ue represents the elastic strain energy, and Ud represents the dissipated strain energy.

When the rock is under triaxial loading, the formulas for calculating each type of energy are as follows:


$$U=\int_{0}^{\varepsilon_{1}}\sigma_{1}d\varepsilon_{1}+\int_{0}^{\varepsilon_{2}}\sigma_{2}d\varepsilon_{2}+\int_{0}^{\varepsilon_{3}}\sigma_{3}d\varepsilon_{3}$$ (2)

$$U_{e}=\frac{1}{2E}\left[\sigma_{1}^{2}+\sigma_{2}^{2}+\sigma_{3}^{2}-2v(\sigma_{1}\sigma_{2}+\sigma_{2}\sigma_{3}+\sigma_{1}\sigma_{3})\right]$$ (3)

In the formula, σ1, σ2, and σ3 represent the external stresses applied to the material in the three directions, while ε1, ε2, and ε3 represent the strains generated by the stresses. E and v are the elastic modulus and Poisson’s ratio of the sample, respectively. It is worth noting that in a uniaxial compression test, there is no constraint, which means σ2 = σ3 = 0. This simplifies equations (2) and (3) to the following forms:


$$U=\int_{0}^{\varepsilon_{1}}\sigma_{1}d\varepsilon_{1}$$ (4)

$$U_{e}=\frac{\varepsilon_{e}\sigma_{1}}{E}$$ (5)

In the formula, εe represents the elastic strain.

**Fig 18:**
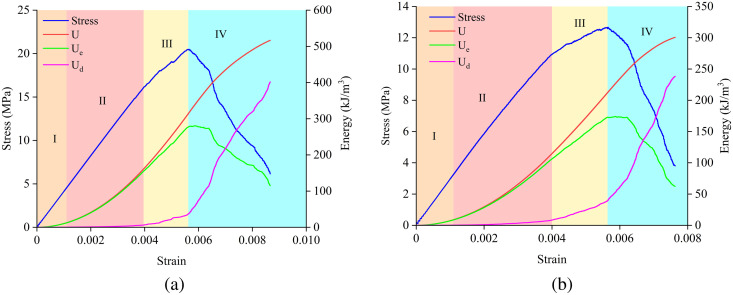
Energy evolution processes: (a) *α* = 0°, L = 20 mm; (b) *α* = 45°, L = 30 mm.

Fig 18 shows the energy evolution processes for samples with α = 0°, L = 20 mm and α = 45°, L = 30 mm. From the figure, it can be seen that the energy evolution undergoes four stages: the initial energy stage (Stage I), the elastic energy storage stage (Stage II), the accelerated energy dissipation stage (Stage III), and the post-peak energy release stage (Stage IV). The ratio of elastic strain energy to input strain energy and the ratio of dissipated strain energy to input strain energy can be used to characterize the elastic deformation and plastic failure degree of the sample, respectively (k_e_ = U_e_/U, k_d_ = U_d_/U).

Since the particles in the numerical model are rigid bodies, there is no initial compaction stage, and the strain energy is basically zero at the initial stage of loading. After entering the linear elastic deformation stage, the input energy in the sample is mainly stored in the form of elastic strain energy, while the dissipated energy remains basically unchanged, causing ke and U to increase approximately linearly with the increase in axial strain. The energy in this stage is mainly used for the reversible elastic deformation of the sample, with only a small number of new microcracks generated. When the stress approaches the peak stress, the microcracks generated in the interlayer region of the sample require dissipation of strain energy, causing the elastic strain energy to reach its limit and then start to release. Most of the input energy U is converted into dissipated energy Ud. At this point, the energy ratios ke for the samples with α = 0°, L = 20 mm and α = 45°, L = 30 mm are 87.56% and 82.13%, respectively, and kd are 12.44% and 17.87%, respectively. After the peak stress, the internal damage of the sample gradually intensifies, and the microcracks continuously propagate and gradually coalesce to form macroscopic cracks, eventually leading to the failure of the sample. During this process, the accumulation of elastic energy in the specimen begins to decrease rapidly and reaches its minimum, while the dissipated energy transitions from slow growth to rapid increase. At this point, ke for the samples with α = 0°, L = 20 mm and α = 45°, L = 30 mm decreases by approximately 65.32% and 71.37%, respectively, and kd increases by 65.32% and 71.37%, respectively. It can be concluded that the energy dissipation of the sample is mainly concentrated in the post-peak stage and is closely related to the propagation and coalescence of microcracks within the rock mass.

### 4.6 Failure modes of samples under different α and L

**Fig 19:**
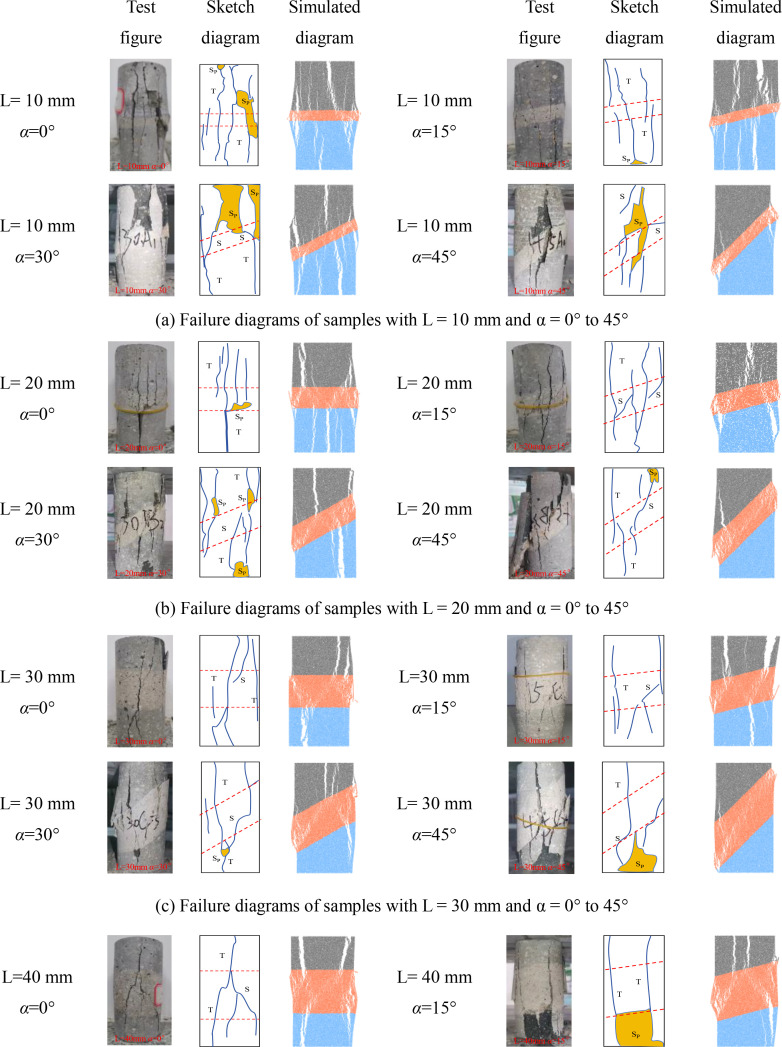
Final failure diagrams of composite rock mass samples with weak interlayers of different inclination angles and thicknesses.

Fig 19 shows the final failure diagrams from experiments and numerical simulations for samples with different interlayer inclination angles (α) and thicknesses (L). In the crack sketches shown in the figure, T represents tensile cracks, S represents shear cracks, and Sp represents spalling surfaces. It can be seen from the figure that although the failure patterns of samples with weak interlayers of different inclination angles and thicknesses vary, the initial failure of all samples is concentrated at the weak interlayer or at the interface between layers. The final failure is mainly characterized by the overall failure of the weak interlayer sample, meaning that the failure characteristics obtained from the experiments are basically consistent with the numerical simulation results.

For samples with interlayer inclination angles α of 0° and 15° and thicknesses of 10 mm and 20 mm, cracks first initiate within the weak interlayer or at the interface between layers, then propagate towards both ends, and finally form one or more cracks caused by tensile action, leading to tensile failure of the sample without significant interlayer slip. It can be concluded that the failure of the sample is mainly controlled by tensile cracks, and the failure mode is predominantly tensile failure. As the interlayer thickness increases to L = 30 mm and 40 mm, the thicker interlayer disperses the stress within the rock mass, leading to enhanced local shear stress and a more complex crack propagation path. Although the number of visible cracks in the sample gradually decreases, and the cracks gradually transition from tensile cracks to shear cracks, the failure characteristics of the sample at this stage are still controlled by tensile cracks. It can be inferred that when the interlayer inclination angle is small, although the number of different types of cracks changes, it does not affect the final failure mode of the sample.

Similar to samples with interlayer inclination angles α of 0° and 15°, the initial cracks in samples with interlayer inclination angles of 30° and 45° still initiate within the interlayer or at the interface between layers and then propagate along the inclination direction of the weak interlayer. At the same time, spalling of the surface rock occurs at the junction between the hard rock and the interlayer. For samples with interlayer thicknesses of 10 mm and 20 mm, the interlayer slip phenomenon is not significant, and local shear failure occurs with relatively weak shear action. The overall failure mode of the sample is mainly a combination of tensile and shear failure. When the interlayer thickness increases to 30 mm and 40 mm, although the cracks in the hard rock part of the sample are still mainly tensile cracks, the shear slip action of the sample becomes more significant as the cracks propagate, which to some extent weakens the spalling degree of the hard rock. In addition, due to the interface slip action provided by the weak interlayer structure, shear cracks dominate overall, and the failure mode of the sample gradually transitions from tensile failure to shear failure, ultimately becoming predominantly shear failure. Table 3 summarizes the three typical failure modes of samples with different interlayer inclination angles (α) and thicknesses (L), including predominantly tensile failure, predominantly shear failure, and a combination of tensile and shear failure. In the crack sketches, V represents V-shaped cracks. Additionally, since the strength of soft rock is lower than that of hard rock, its crack development degree is significantly higher than that of hard rock.

**Table 3:** Classification of failure modes for the samples.

Failure mode	Crack propagation type	Schematic diagram of failure type	Description of crack propagation type	Sample
Mainly tensile failure	Ⅰ	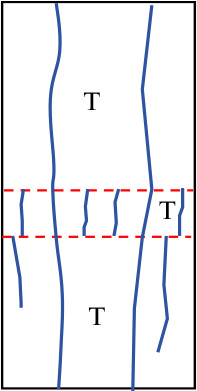	Vertical tensile cracks appear between the hard rock and the weak interlayer	*α* = 0° ~ 15°, L = 10 mm ~ 40 mm
	Ⅱ	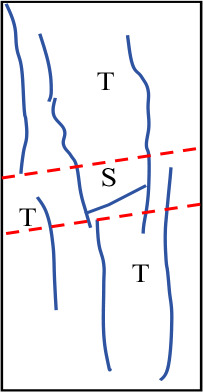	Vertical tensile cracks appear between the hard rock and the weak interlayer, with a small number of shear cracks occurring in the interlayer region	
	Ⅲ	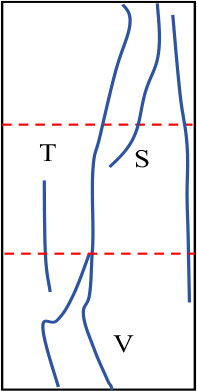	V-shaped cracks appear between the hard rock and the weak interlayer, with a small number of shear cracks occurring in the interlayer region	
Mixed tensile-shear failure	Ⅳ	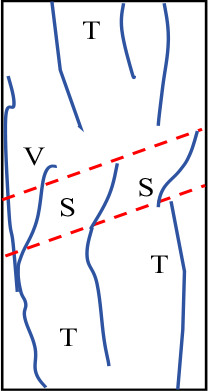	The hard rock is filled with tensile cracks, while the weak interlayer is filled with shear cracks and a small number of tensile cracks	*α* = 30° ~ 45°, L = 10 mm ~ 20 mm
Mainly shear failure	Ⅴ	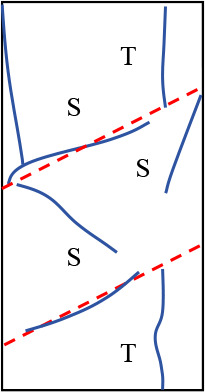	Slippage occurs at the interface between layers	*α* = 30° ~ 45°, L = 30 mm ~ 40 mm

## 5 Discussion

Based on the assumption that the sample is a homogeneous body, this paper investigates the complex mechanical properties and failure behaviors of composite rock masses with weak interlayers. The stress-strain, acoustic emission ringing counts, stress fields, energy, and crack evolution characteristics of samples with weak interlayers of different inclination angles and thicknesses are analyzed. However, in actual engineering rock masses, weak interlayers often contain fissures, defects, etc., which make them exhibit heterogeneous characteristics. The applicability of the research results based on the homogeneous body assumption needs further exploration. Therefore, relevant studies are conducted on composite rock masses with weak interlayers containing fissures of different inclination angles to investigate the mechanical properties and failure characteristics of heterogeneous composite rock masses with fissured weak interlayers.

In this section, taking a sample with a weak interlayer inclination angle (α) of 0° and a thickness (L) of 20 mm as an example, fissures with inclination angles of 0°, 30°, 45°, 60°, and 90° are prefabricated in the middle of the weak interlayer. The angle between the fissure and the horizontal direction is denoted as β. Through laboratory rock mechanics experiments, the mechanical properties and failure modes of composite rock masses with weak interlayers containing fissures of different inclination angles are focused on. To ensure the reliability of the experimental results and compare them with numerical simulation results.

### 5.1 Analysis of mechanical properties and failure characteristics

**Fig 20:**
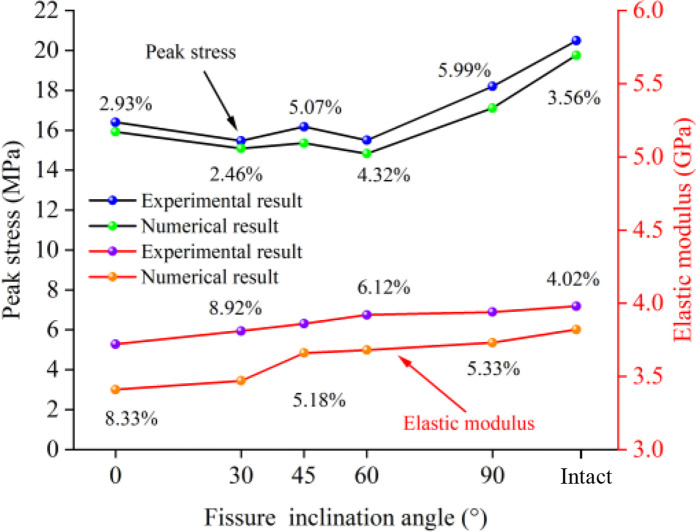
Comparison of peak stress and elastic modulus between numerical simulation and laboratory test results for fissured composite rock masses.

**Fig 21:**
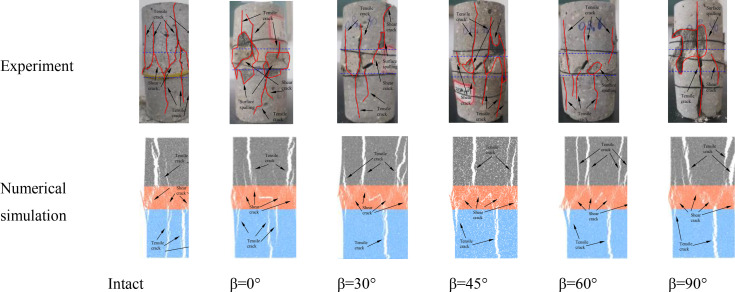
Comparison of failure modes between numerical simulation and laboratory test results for fissured composite rock masses.

Fig 20 shows the comparison between the numerical simulation results and laboratory test results for samples with different fissure inclination angles and intact samples. It can be seen from Fig 20 that the macroscopic mechanical parameters of the fissured composite rock mass are significantly lower than those of the intact sample, and the peak stress exhibits a “W”-shaped variation trend with the increase in fissure inclination angle, while the elastic modulus shows a gradually increasing trend. The peak stress of the intact sample is 2.29 MPa to 5.02 MPa higher than that of the fissured samples, and the elastic modulus is 0.04 GPa to 0.26 GPa higher. This indicates that the presence of fissures weakens the mechanical properties of the composite rock mass, reduces the peak stress and elastic modulus, making the strength of fissured rock lower than that of intact rock. It can be seen from Fig 21 that the final failure of the fissured weak interlayer sample is caused by the intersection and coalescence of microcracks within the sample and cracks generated at the tips of the prefabricated fissures. The cracks are mainly concentrated in the weak interlayer region, manifesting as a large number of tensile cracks and some shear cracks developing around the fissures, along with severe spalling on both sides of the fissures. This is basically consistent with the failure characteristics of intact weak interlayer rock masses, which are predominantly tensile failures.

### 5.2 Error analysis

From the comparative analysis of the experimental results and numerical simulation results, it can be seen that there is a certain degree of error between the peak stress, elastic modulus, and crack propagation characteristics. This is mainly because the composite rock mass with a weak interlayer is made by natural cementation, which gives the interface a certain bond strength under low stress. However, the two-dimensional numerical model interface established using PFC2D typically adopts a parallel bond model, which is difficult to fully simulate the continuous cementation effect [31]. The samples prepared in the experiment are three-dimensional cylinders, and the two-dimensional numerical model cannot fully consider the stress distribution and local deformation constraints in the thickness direction, which is also one of the reasons for the slight error between the two [32]. However, the two-dimensional model can well simulate the failure mechanics behavior of rock, and the two-dimensional numerical simulation results can to some extent reflect the three-dimensional problems [33]. In addition, the natural inhomogeneity of the test materials and the randomness of particle distribution in the numerical simulation are also reasons for the error between the two.

### 5.3 Engineering failure analysis

**Fig 22:**
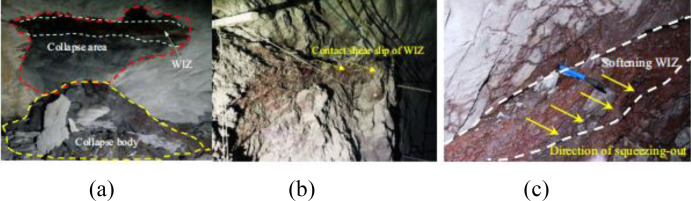
Chamber failure diagrams (a: rock mass collapse, b: local contact shear slip, c: plastic extrusion failure of chamber sidewall) [34].

During the excavation of engineering rock masses containing weak interlayers, the original stress equilibrium will be disrupted, often accompanied by large deformations and crack propagation, which in turn leads to a series of engineering rock mass failure problems. For example, local shear slip failures and plastic extrusion failures that have occurred in previous actual projects (see Fig 22) [34,35], these two important issues can be explained to some extent by the failure characteristics of rock masses with weak interlayers (Figs 12, 15, 19). The on-site failure results indicate that rock masses with weak interlayers exhibit tensile-shear mixed failure during underground excavation, with plastic extrusion failure being dominated by tensile fractures, followed by shear fractures [36,37].

For samples with interlayer inclination angles of 30° and 45° and thicknesses of 10 mm and 20 mm, the interlayer slip phenomenon is not significant, and local shear failure occurs with relatively weak shear action. The overall failure mode of the sample is mainly a combination of tensile and shear failure. When the interlayer thickness increases to 30 mm and 40 mm, the shear slip action of the sample becomes more significant as the cracks propagate. Influenced by the interface slip action provided by the weak interlayer structure, shear cracks dominate overall, and the failure mode of the sample gradually transitions from tensile failure to shear failure, ultimately becoming predominantly shear failure. This is to some extent consistent with the local shear slip failure observed in on-site interlayer rock masses. In the experiment, for samples with interlayer inclination angles (α) of 0° and 15° and thicknesses ranging from 10 mm to 40 mm, cracks first initiate within the weak interlayer or at the interface between layers and then propagate towards both ends, ultimately leading to tensile failure of the sample without significant interlayer slip. The failure of the sample is mainly controlled by tensile cracks, and the failure mode is predominantly tensile failure, which is consistent with the plastic extrusion failure observed in on-site interlayer rock masses. It can be concluded that the failure modes of rock masses with weak interlayers of different inclination angles and thicknesses revealed in this study can provide certain guidance for actual on-site engineering projects.

## 6 Conclusions

(1) The strength of rock masses containing weak interlayers lies between that of intact hard rock and soft rock. Both the uniaxial compressive strength and elastic modulus decrease with the increase in the thickness and inclination angle of the weak interlayer. The thickness of the interlayer has a greater impact on the elastic modulus, while the inclination angle of the interlayer is more sensitive to the peak strength.
(2) Under compressive loading, the contact forces and displacements between rock particles first increase and then decrease, and the stress concentration area first forms within the weak interlayer. The energy absorbed by the rock mass is mainly dissipated through the propagation of microcracks in the interlayer region, and the energy dissipation mainly occurs in the post-peak stage, during which the proportion of dissipated energy increases by nearly 70%.
(3) The failure of the sample initiates within the weak interlayer and at the interface between layers and ultimately ends with the joint failure of both soft and hard rock masses. Microcracks initiate and develop extensively within the weak interlayer and then propagate towards the hard rock region along the loading direction. An increase in the thickness of the interlayer stabilizes the structure, and the failure is mainly dominated by tensile splitting; an increase in the inclination angle promotes a transition of the failure mechanism to shear dominance.

## Supporting information

S1 Data Fig 4 Data. (XLSX)

S2 Data Fig 6 Data. (XLSX)

S3 Data Fig 7 Data. (XLSX)

S4 Data Fig 8 Data. (XLSX)

S5 Data Fig 11 Data. (XLSX)

S6 Data Fig-12 Data. (XLSX)

S7 Data Fig-14 Data. (XLSX)

S8 Data Fig 18 Data. (XLSX)
